# Comment on: intraocular lymphoma masquerading as unilateral hypopyon anterior uveitis: a case report

**DOI:** 10.1186/s12348-023-00324-7

**Published:** 2023-02-28

**Authors:** Cem Evereklioglu, Hidayet Sener, Osman Ahmet Polat, Fatih Horozoglu

**Affiliations:** Department of Ophthalmology, Division of Uvea-Behçet Unit, Erciyes University Medical Faculty, Kayseri, Türkiye

**Keywords:** ALL, Color, Hypopyon, Neoplastic, Precipitation, Pseudohypopyon, Sediment, Tumoral

Dear Editor,

We have read the very recent case report of Zamani et al. entitled “Intraocular lymphoma masquerading as unilateral hypopyon anterior uveitis: a case report” published in the Journal of Ophthalmic Inflammation and Infection in 2022 [1]. The authors reported a 55-year-old woman with large B cell lymphoma who had recurrent, refractory, unilateral anterior chamber hypopyon. Her visual acuity was decreased (20/32) for the past 2 months with normal intraocular pressure. The authors described the ocular finding as “hypopyon anterior uveitis” and made various tests and work to exclude possible etiologies including Behçet disease and ankylosing spondylitis, which revealed all normal results.

The case of the authors is most timely for the differential diagnosis of “hypopyon” and “pseudohypopyon”, particularly for a prompt diagnosis of tumoral masquerading syndromes. However, we have a few observations on the anterior chamber precipitation and suggestions to make it a remarkable read for the assistance of oncologists and ophthalmologists interested in ocular complications of lymphoma or leukemia. Indeed, Fig. 1 of the presented woman does give objective initial clues for performing a true definition of the anterior chamber sediment and differential diagnosis.

**Fig. 1:**
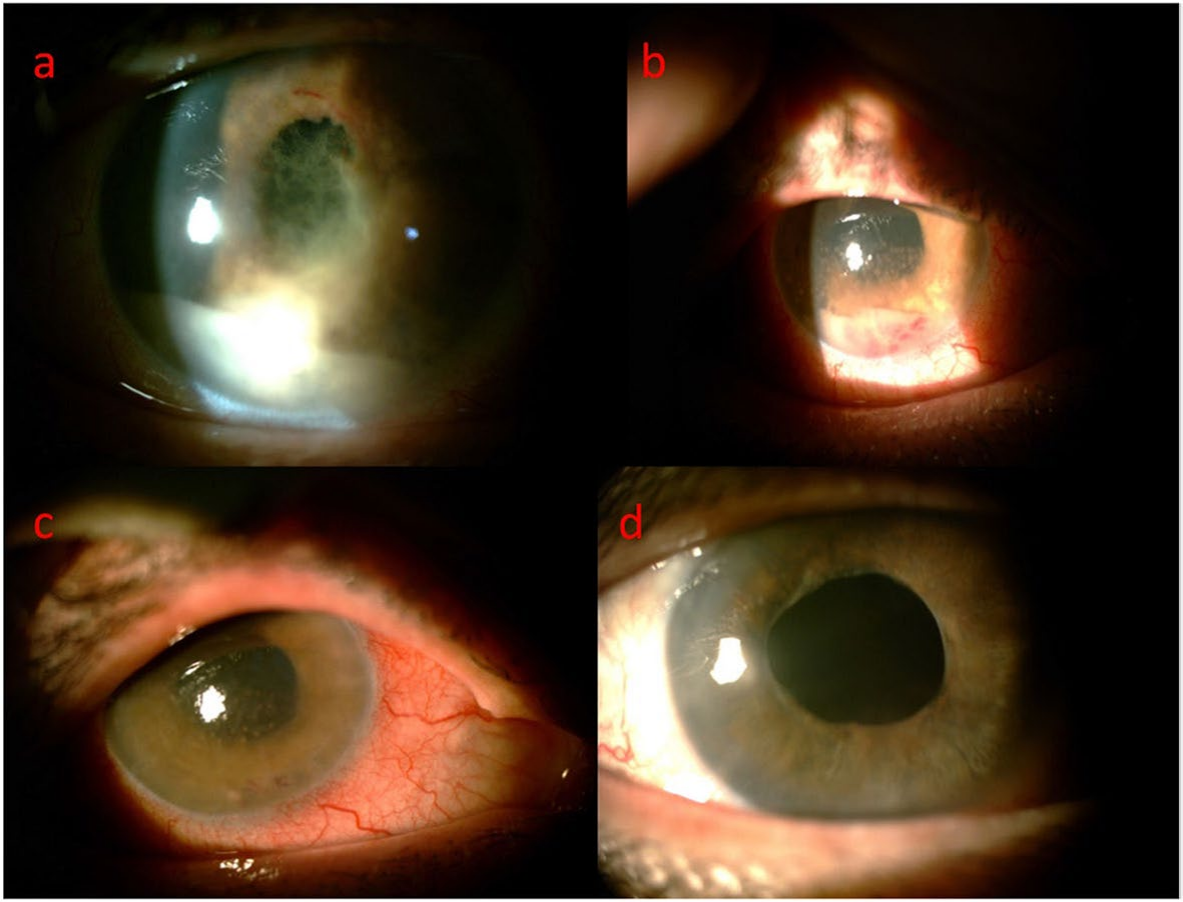
Slit photography of the right eye; hypopyon anterior uveitis with severe fibrinous reaction following 2 months therapy as a uveitis case (**a**), Following third ocular sampling and cataract surgery (**b**), two weeks following intraocular methotrexate injection (**c**), One year after the first presentation (**d**)

*First,* the shape of the uveitic collections in the anterior chamber is pathognomonic, which results in horizontally layered white meniscus formation typically heaped up centrally rather than at its edges. The ocular presentation of the woman in Fig. 1a is typically a layered hypopyon as the authors indicated. However, when we check Fig. 1b carefully, the anterior chamber precipitation is heaped up at its edges (not centrally), especially on its nasal site. Such a macroscopic external finding to the naked eye should have forced the authors to make the diagnosis of “pseudohypopyon” from neoplastic origins at this stage. Therefore, the term “hypopyon”, which was stated throughout the text, does not seem to be correct for the description of this collection. Strictly speaking, the more appropriate nomenclature should be “neoplastic pseudohypopyon” for such a meniscus in Fig. 1b that strongly indicates “masquerade syndromes of malignancy”. Indeed, such anterior chamber collections contain neoplastic cells, like the sampling of authors confirmed large B cell lymphoma, which was positive for CD19 and CD20 [1]. On the other hand, it should be taken into account that repetitive head shaking or chronicity of the disease may cause fibrinoid anterior chamber reaction and severe hypopyon uveitis due to uveal tissue irritation from tumoral cell irritation, like in Fig. 1a.

*Second,* Fig. 1b demonstrates that the eye has no ciliary injection. Indeed, intraocular inflammation traditionally presents with severe ciliary hyperemic injection or congestion with violaceous hue [2]. The perilimbal area of the involved right eye of the woman seems to be white, not dusky red, indicating the meniscus to be a “pseudohypopyon” for physicians.

*Third,* the differential diagnosis between “hypopyon” and “pseudohypopyon” is very important for clinicians since their managements and prognoses are entirely different [3]. Indeed, the behavior of anterior chamber collection may be an indicator of some specific disorders. Therefore, our next question to Zamani et al. is “did they assess the mobility of the precipitation in Fig. 1b?” That is, we wonder whether the collection in the anterior chamber was shifting or non-shifting upon posture change or head-tilting? It is known that the etiology of the meniscus affects its mobility at various speeds [3]. Actually, “tumoral pseudohypopyons” are mobile precipitations and dislocate into another part of the eye *within seconds* upon head-tilting or leaning to one side, which is a pathognomonic observation called “shifting/mobile pseudohypopyon”. So, if the authors had changed the position of the woman to one side, they would possibly have encountered the shifting feature of the collection. On the other hand, hypopyon from uveitic etiologies has “non-shifting” characteristics, which are totally immobile, possibly like in Fig. 1a.

*Finally,* the color of the anterior chamber sediment can direct oncologists and ophthalmologists to a certain etiology [4]. Zamani et al. stated in their case report that the anterior segment showed 0.2 mm hypopyon with keratic precipitates in Fig. 1a [1]. However, the level of hypopyon is actually about 2 mm in height. In addition, the authors did not refer to Fig. 1b in the text, which has several clues indicating its pseudohypopyon appearance as stated above. If we carefully check Fig. 1b again, the “blood-tinged” pinkish nature of the gray collection can easily be recognized, which signifies spontaneous hyphaemia due to possible malignancy [5]. Therefore, such anterior chamber precipitation should be defined as “blood-streaked, pinkish-colored pseudohypopyon”, not hypopyon. Indeed, vitreoretinal ocular lymphoma or leukemia causes spontaneous, “blood-streaked”, tumoral pseudohypopyon consisting of hemorrhagic tumoral cells. However, anterior chamber reaction and secondary severe uveitis with or without keratic precipitates and hypopyon may be seen due to chronic uveal tissue irritation from tumoral cells, as stated above. The authors finally stated in their report that partial response was observed upon topical corticosteroid whereas significant response was observed with the disappearance of “hypopyon” after repeated local chemotherapy using intravitreal methotrexate and rituximab, indicating again the tumoral nature of the pseudohypopyon.

Taken together, if anterior chamber collections are examined with their full characteristics (shape, mobility, color) and perilimbal status, oncologists and ophthalmologists can diagnose (or exclude) intraocular lymphoma (or leukemia) on time and direct such patients to the proper physician for prompt therapy.

## Data Availability

Not applicable.
